# Attitudes and beliefs towards COVID-19 and COVID-19 vaccination among rheumatology patients in a Los Angeles County safety net clinic

**DOI:** 10.1186/s41927-023-00338-7

**Published:** 2023-06-01

**Authors:** Nicole K. Zagelbaum Ward, Suman Pal, Katherine Ruddy, Stavros Savvas

**Affiliations:** 1 Division of Rheumatology, Department of Medicine, Keck School of Medicine, University of Southern California, and Los Angeles County, Los Angeles County + University of Southern California (LAC+USC) Medical Center, Los Angeles, CA USA; 2 Division of Hospital Medicine, Department of Internal Medicine, University of New Mexico Health Sciences Center, Albuquerque, NM 87106 USA

**Keywords:** Coronavirus disease 2019 (COVID-19), Severe acute respiratory syndrome coronavirus 2 (SARS-CoV-2), Rheumatologic disease, Vaccine acceptance, Vaccine hesitancy

## Abstract

**Background:**

The novel Coronavirus disease (COVID-19) pandemic has represented an evolving global threat with high morbidity and mortality. Patients with autoimmune rheumatic diseases and on immune-suppressing medications may be at increased risk to more severe disease, hospitalization, and death. Vaccines are essential to combat the COVID-19 pandemic and curb the spread of infection. Rheumatology patients may be more fearful to receive the vaccine compared to the general population. The Los Angeles County rheumatology patients are primarily Hispanic and represent a unique and possibly particularly vulnerable cohort warranting further exploration into barriers to receive the COVID-19 vaccine. We aimed to explore the willingness of COVID-19 vaccine acceptance among patients with rheumatic disease.

**Methods:**

We conducted a cross-sectional survey to assess the perceptions and barriers to COVID-19 vaccine acceptance in our Los Angeles County rheumatology clinics between July 2021 to September 2021 and received responses from 116 patients.

**Results:**

The majority of respondents were female (83.9%), 41–60 years of age (59.8%), Hispanic (89.2%), with high school or lower level of education (68.7%), and had Rheumatoid Arthritis (56.9%). We found most (88.4%) patients received at least one dose of the COVID-19 vaccine. We identified no differences in vaccine acceptance related to age, education, race, and ethnicity. Most respondents agreed that their health condition puts them at high risk of COVID-19 complications. In addition, individuals reported that they valued being engaged by their rheumatologists in discussions of the risk and benefits of the vaccine prior to receiving it.

**Conclusion:**

We found that the majority of patients were already vaccinated or willing to be vaccinated, at higher levels than general United States population and that a conversation initiated by a rheumatologist can have positive effect on patients’ health behaviors related to COVID-19.

**Supplementary Information:**

The online version contains supplementary material available at 10.1186/s41927-023-00338-7.

## Key points

- COVID-19 vaccine acceptance was relatively high in Los Angeles County patients compared to the national population.
- Vaccine acceptance was not statistically associated with medication use, age, education, race, or ethnicity.
- There was no association between previous COVID-19 infection and willingness to accept the vaccine.
- Dialogue between rheumatology providers and patients is important and may increase vaccine acceptance in the future as booster vaccines and ongoing engagement is critical.

**Supplementary Information:**

The online version contains supplementary material available at 10.1186/s41927-023-00338-7.

## Background

The novel Coronavirus disease (COVID-19) caused by the severe acute respiratory syndrome coronavirus 2 (SARS-CoV-2) was discovered in 2019 and has evolved to represent an evolving global pandemic with over 5 million deaths to date [1]. Highly prevalent health conditions such as obesity, lung disease, cardiovascular disease and diabetes were noted to be associated with increased disease severity and mortality early in the pandemic. However, with lower disease prevalence, the associations of rheumatic disease (RD) with COVID-19 have taken more time to understand in terms of susceptibility to infection as well as disease outcomes. Uncontrolled inflammation in the setting of RD including rheumatoid arthritis (RA), systemic lupus erythematosus (SLE), polymyalgia rheumatica, myositis and vasculitis may put patients at greater risks for contracting the virus as well as increased incidences of morbidity and mortality [2–6]. This may be a result of uncontrolled RD activity, the use of immune suppressing medications, or both. While some studies have shown this relationship more clearly, others have not [6–9].

It is important to protect at-risk individuals from transmission and manifestations of severe complications of disease. Several methods of disease mitigation exist, include social distancing, masking, quarantining, contact tracing, monitoring community transmission, identifying high risk populations and early use of medications. Perhaps of the greatest importance, the development, distribution, and acceptance of the COVID-19 vaccine is crucial to build herd immunity and ultimately combat and control the widespread infection. Barriers to accepting the COVID-19 vaccine in the general population including adverse side effects, worsening of rheumatic disease, and mis-information including impotence and death have been reported [2]. The politization of the COVID-19 pandemic and beliefs surrounding vaccine efficacy has further complicated who may or may not be willing to be vaccinated [10]. This study aimed to further understand barriers to receiving the COVID-19 vaccine among high-risk patients with RD.

## Methods

We conducted a cross-sectional survey among patients visiting rheumatology clinic at Los Angeles County + University of Southern California (LAC + USC) Medical Center for their routine appointments. These patients are primarily economically disadvantaged, Hispanic, speak Spanish as a first language, and are at risk for poorer health outcomes than their insured counterpart. The survey instrument was adapted from previously published study by Shekhar et al. [11]. Two authors with subject matter expertise in RD identified variables of interest and suggested modifications to original survey instrument to assess these variables. The variables of interest included – Rheumatic disease/diagnosis, number of years with disease/diagnosis, current medications. The beliefs around interaction of COVID-19 were also considered of interest and assessed through addition of following statements to the Likert agreement scale part of original questionnaire – “ My medical condition places me at high risk of COVID-19 complication”; “My medications place me at high risk of COVID-19 complications”; “Getting the COVID-19 vaccine will worsen my medical condition or cause me to flare”; “If my rheumatologist recommends the vaccine, I will be more likely to get it.” Questions from the original survey instrument which were not considered relevant to current population were omitted. The modified survey was reviewed by third author with content knowledge of COVID-19 vaccine acceptance research. Further modifications for language clarity were made by mutual consensus. The survey instrument was available in both English and Spanish. Spanish version of survey instrument was reviewed by authors with native proficiency in Spanish for language clarity and readability. The survey instrument was made available as a paper form and was self administered. All responses were anonymized. Responses were collected July through September 2021. The study was approved by Institutional Review Board of USC (#HS-16 00,977, Improving Quality of Care in Rheumatic Disease).

### Sampling

Consecutive patients visiting the clinic were assessed for eligibility. Adult patients > 18 years of age and with a previously diagnosed RD were included in the study. Informed consent was obtained prior to participation in the study. Exclusion criteria included patients who did not speak English or Spanish, patients who are illiterate, inability or unwillingness to participate in the study or fill out the survey.

### Analysis

The primary outcome of the study was receipt of COVID-19 vaccine. This was assessed by the confirmation of the statement, “I have received the COVID-19 vaccine already.” Based on response, respondents were divided into two groups – vaccine hesitant and vaccine non-hesitant. Descriptive analysis was done using frequency distribution for demographic variables of gender, age, ethnicity, race, size of household (number of individuals living with you in primary residence), and education. Frequency distribution was also used to identify disease specific factors such as primary RD, and number of years living with disease. Univariate analysis was used to identify association of vaccine hesitancy with demographic variables collected such as age, gender, racial or ethnic identity, education level, primary diagnosis, medication regimen, source of information, etc. Chi-square test was used to assess for significance of association, Fisher exact test was used instead when cell populations were below 5. Given the large number of comparisons made, Bonferroni correction was used to correct for family wise error rate and a p-value of < 0.05/12, i.e. p-value of < 0.004 was considered significant.

Attitude of respondents toward COVID-19 vaccines were recorded as degree of agreement with statements on a Likert scale. Descriptive analysis with frequency distribution was used to assess the results and data was presented in graphical form.

## Results

One hundred and sixteen respondents completed the survey. In the literature, response rates have been identified to be highly variable based on type of survey, follow up, geography and interview type [12]. Due to the nature of our clinic, mixed types of visits (phone, video and in person) as well as language barriers, the number of requested surveys could not be recorded and therefore we cannot report a calculated response rate. Three respondents did not answer the questions for the primary outcome and one response did not provide age to verify meeting inclusion criteria, therefore these responses were excluded from analysis. One hundred and twelve responses were included in analysis.

The majority of respondents were female (83.9%), 41–60 years of age (59.8%), Hispanic (89.2%), and with high school or lower level of education (68.7%). More than half of respondents had RA. The majority (72.3%) had been diagnosed with RD five or more years ago. About half (51.8%) noted that a doctor had discussed COVID-19 vaccine with them.

Only 13 patients (11.6%) had not received COVID-19 vaccine in our study population. We identified no statistically significant association of vaccine hesitancy associated with age, gender, education, racial identity, ethnicity, rheumatological diagnosis, current medications, or duration since diagnosis (Table 1). Personal experience with COVID-19, discussion with a doctor regarding COVID-19 vaccine and primary source of information did not show any statistically significant associations with vaccine hesitancy.

**Table 1:** The age

Variable	Non hesitant group (n = 99)	Hesitant group (n = 13)	*P*-value*
Gender			
Male	16	1	0.67
Female	82	12	
Transgender/nonbinary	1	0	
Age (yr.)			
< 18	0	0	
18–30	8	2	0.58
31–40	9	0	
41–50	35	4	
51–60	23	5	
61–70	19	2	
> 70	5	0	
Ethnicity			
Hispanic	87	13	0.68
Non-Hispanic	7	0	
Not reported	1	0	
Don't wish to reply	2	0	
Missing	2	0	
Racial identity			
Native American	2	0	0.21
Asian, Native Hawaiian, Pacific Islander	4	0	
Black	3	0	
White	21	0	
More than one	5	1	
Unknown	21	6	
Other	16	4	
Missing	26	2	
Highest level of formal Education			
No formal	7	0	0.98
Some formal	27	4	
High school	34	5	
Some college credit	12	1	
Trade/technical	5	1	
Bachelor	8	1	
Masters	1	0	
Professional	1	0	
Doctorate	0	0	
Missing	5	1	
Personal experience with COVID-19			
I had Covid	10	1	0.96
Family member had COVID	14	2	
Someone I know personally had COVID	20	2	
No one I know personally had COVID	36	4	
Missing	7	2	
Primary Rheumatological disease			
Rheumatoid arthritis	58	8	0.47
SLE	5	1	
Scleroderma	2	1	
Sjogren	2	0	
Vasculitis	1	0	
Ankylosing Spondylitis	1	1	
Others	3	0	
Do not wish to reply	2	1	
Missing	7	1	
Years with disease			
1 yr	9	2	0.92
1–5 years	18	3	
5–10 years	22	2	
10–20 years	23	3	
> 20 years	20	3	
Missing	8	0	
Medication			
A doctor has discussed COVID vaccine with me			
Yes	52	6	0.51
No	42	7	
Missing	6	0	
Language			
Spanish	66	11	0.19
English	33	2	
Medication			
Prednisone	17	3	0.49
HCQ	30	2	
MTX	24	4	
Mycophenolate	7	3	
Azathioprine	3	0	
Tofacitinib	4	0	
Injection	18	2	
Infusion	12	1	
Others	10	3	
None	0	0	
Primary source of information for COVID-19			
Television	56	6	0.30
Internet	40	5	
Social media	31	1	
Friends and/or family	32	2	
Church	8	0	
Doctor’s office	24	1	

*Chi-square test used when cell values > 5, If cell values < 5 Fisher exact test was used

*SLE* systemic lupus erythematosus, *HCQ* hydroxychloroquine, *MTX* methotrexate

**Fig. 1:**
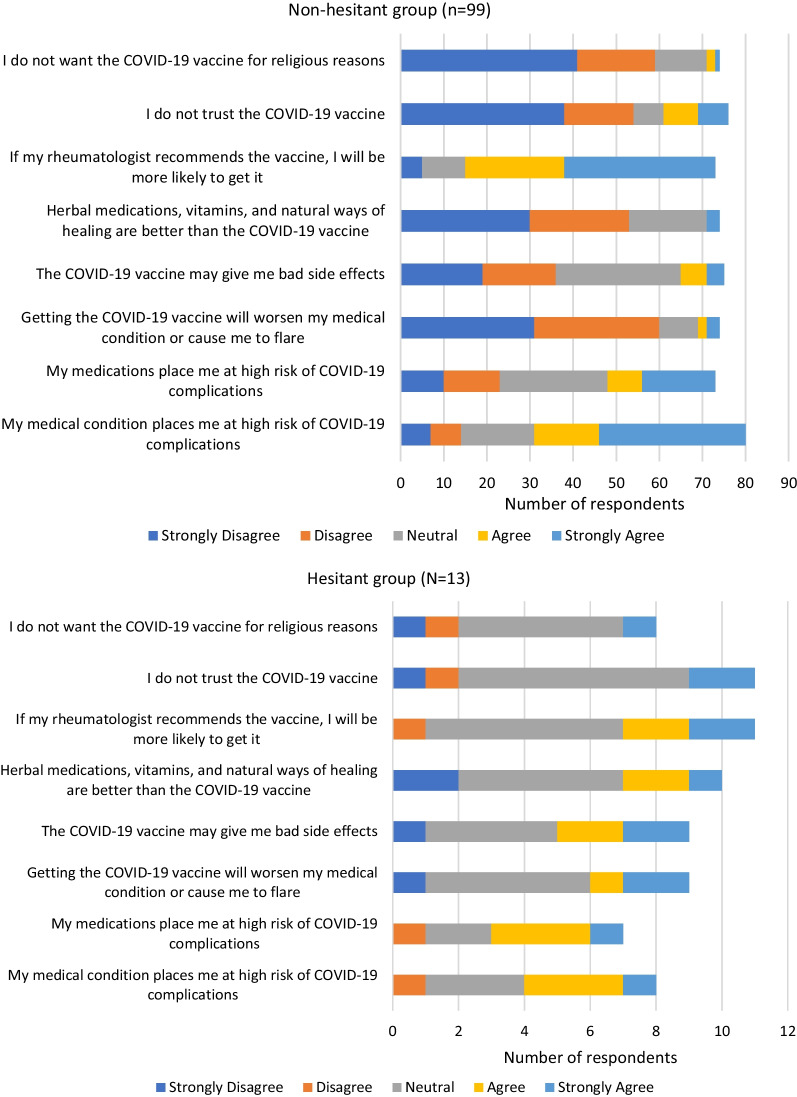
Attitude of vaccine hesitant and non-hesitant group toward COVID-19 vaccine

When assessing patient attitudes and beliefs, we note certain trends. A majority of respondents in both groups believe that their medical condition puts them at high risk of COVID-19 complications. In additional, a majority of respondents in vaccine hesitant group also believe that their medications puts them at high risk of COVID-19 complications. However, the two groups tend to differ in beliefs regarding key misconceptions about COVID-19 vaccine with the vaccine non-hesitant group disagreeing or strongly disagreeing that COVID-19 vaccines will worsen medical condition or cause flares, that COVID-19 vaccine will give bad side effects or that they do not trust the COVID-19 vaccine. For the same statements, the vaccine hesitant group were mainly neutral or in agreement with the statements. Most respondents in both groups reported that they value being engaged by their rheumatologists in discussions of the risk and benefits of the vaccine prior to receiving it, though a greater proportion of respondents in the vaccine non-hesitant group agree that a rheumatologist’s recommendation would make it more likely to receive the vaccine (Fig. 1).

To assess whether our results were affected by choice of our outcome variables, we performed a post-hoc sensitivity analysis by converting the following variables into a binary distribution – Age < 40yrs vs > 40yrs, Education – Up to high school (No college) vs some college or more, Personal experience with COVID–None vs any (had COVID, family member had COVID, or someone I know personally had COVID). The change in outcomes did not change the results of our analysis and we did not note any statistically significant association with these revised outcome variables (Additional file 1).

## Discussion

The current study demonstrates that RD patients of a Los Angeles County outpatient rheumatology clinic represent a cohort of patients that may be willing to be vaccinated at higher rates than the general population and possibly when compared to other cohorts with RD. To date, 75.1% of residents are vaccinated in Los Angeles County overall [13]. This is compared to the 88.4% acceptance rate amongst our patients. Rates of vaccination in the adult population in Los Angeles County have not been reported. Although the rate of vaccination was high, it is unclear why this population may be more willing to receive the vaccine when compared to general population. Some potential explanations may be that our survey includes a self-selected group of engaged rheumatology patient who may be more interested in their health and health-related outcomes. In addition, these rheumatology patients are on immune-suppressing medications, many of which have recommended vaccine for other pathogens such as influenza, pneumococcus, varicella among others. Therefore, they may be more willing to accept vaccines in general as a result. In addition, the Los Angeles community is diverse, primarily democratic, and may be more engaged in their health and the prevention of disease when compared to other populations [14, 15].

Our findings stand in contrast with some current literature on barriers of vaccination among patients with RD. Several studies exist which explore the relationship between COVID-19 vaccine hesitancy and patients with RD. Gaur et al. surveyed 280 patients in a rheumatology clinic in Western India and found 152 (54%) reported willingness to be vaccinated [2]. They found older age and higher education level to be significant factors associated with vaccination and promoted vaccine educated as possible intervention that is worthy of further exploration. Priori et al. surveyed RD patients in the first few weeks of the release of the COVID-19 vaccine and found that 344 (54.9%) of 626 patients were willing to accept the vaccine [16]. They also found that RD patients were less willing to accept the COVID-19 vaccine when compared to controls. El Kibbi et al. surveyed 1,594 patients in 19 Arab countries including Algeria, Bahrain, Egypt, Iraq, Jordan, Syria and United Arab Emirates and found only 29% of patients to be vaccinated compared to 59% of controls who were healthcare professionals [17]. Yurtass et al. conducted a web-based questionnaire in Egypt which included 731 RD patients and found that 29.2% of patients were willing to be vaccinated which was comparable to the general population and significantly less willing than healthcare workers [18]. Sadun et al. surveyed over 14,000 patients in the United States and ultimately had 433 patients respond and found 85% of patients were willing to accept the vaccine [19]. They identified that vaccine acceptance was associated with white race, Democratic party, higher income and older age.

A summary of the literature on this topic to date is outlined in Table 2. The only other survey conducted in an academic center in the United States [20] found similar results to ours. Some potential explanations behind of the discrepancies in these results include cultural differences, political affiliation, access to vaccine, ability to visit doctors’ offices, educational resources, and patients’ ability to take time away from work and childcare support to be able to attend a medical appointment to receive the COVID-19 vaccine. Our study is unique because we report a primarily underserved, Hispanic community of Los Angeles which is worthwhile to add to the existing body of literature.

**Table 2:** Summary of Trials COVID-19 vaccine in autoimmune and rheumatic disease patients

Study	Design	Participants	Location	Comparator groups	Main findings
COVID‑19 vaccine hesitancy in patients with systemic autoimmunerheumatic disease: an interview‑based survey [2]	Cross sectional interview based survey	280	Western India	102 controls	Vaccine acceptance reported in 54% of RD patients. Patients > 45 years of age were more willing than those age < or equal to 45 years
SARS-CoV-2 vaccine hesitancy among patients with rheumatic and musculoskeletal diseases: a message for rheumatologists [16]	Cross sectional interview based survey	830	Rome, Italy	370 controls	No difference among patients RD diagnosis subgroups
Acceptability of the COVID-19 Vaccine in Patients with Rheumatic Diseases and Healthcare Professionals in 19 Arab Countries [17]	Cross sectional web-based survey	1,594	19 Arab countries including Algeria, Bahrain, Egypt, Iraq, Jordan, Syria and United Arab Emirates	1,517 healthcare professionals	Acceptance to receive COVID-19 vaccine was reported by 344 (54.9%) of 626 patients with rheumatic and musculoskeletal disease, 29% of the patients were vaccinated compared to 59% of healthcare professionals
Willingness to get the COVID-19 vaccine among patients with rheumatic diseases, healthcare workers and general population in Turkey: a web-based survey [18]	Cross sectional web-based questionnaire survey	732	Istanbul, Turley	763 individuals from general population, 320 hospital workers	29.2% of patients with RD were willing to be vaccinated, compared to 34/6% of general population and 52.5% of hospital workers
COVID-19 Vaccine Hesitancy in Rheumatology Patients [20]	Cross sectional electronic survey	433	Southeastern United States	None	Rheumatology patients wanted to be vaccinated against COVID-19 at a rate that was higher than national surveys estimates at the time (85% vs 60–69%)

The data regarding response to messenger ribonucleic acid (mRNA) vaccination amongst RD patients has been mixed, with one study demonstrating about one third of patients with low immune response [21]. This report noted that the vaccine may also precipitate flare, which was found in about 11% of patients [21]. It is important to note in our cohort that medications (type or total number) did not play a significant role in respondents’ willingness to be vaccinated. In the current literature, it is understood that certain medications may put patients at a higher risk for complications secondary to the virus [8]. For example, B cell depleting therapies including rituximab, cyclophosphamide, high doses prednisone and sulfasalazine may be associated with higher incidence of hospitalizations and complications of COVID-19 pneumonia and acute respiratory distress syndrome [22]. Other medications including hydroxychloroquine, leflunomide, methotrexate, tumor necrosing factor inhibitors, low dose prednisone (< 10 mg daily) may not have the same association [23]. This important topic with an evolving body of literature highlights the importance of close communication between patients and their rheumatologist.

Our study had limitations. Individuals who filled out the survey were self-selected, willing to fill out the voluntary questionnaire and therefore may represent a more engaged group of RD patients and partially explain our high rate of vaccine acceptance. While it would have been noteworthy to include patients who do not routinely come to rheumatology clinic, are not engaged in their healthcare and disease prevention, and were too sick to come to their rheumatology appointments, this was outside of the scope of patients able to be surveyed in our study. Some surveys were missing data, which was addressed in the discussion outlined above. Our sample size was relatively small and therefore larger studies with control groups may provide greater insight and be able to obtain statistically significant data which our study did not.

## Conclusions

We report a cohort of RD patients and outline potential barriers to receiving COVID-19 vaccination. Our study stands in contrast of several international surveys outlined in this paper, which found that vaccine acceptance rate in RD patients is lower than the general population. Our findings are in agreement with another survey conducted in the United States, however we include a different demographic that is primarily Hispanic and socioeconomically disadvantaged who may be more at risk of poor outcomes related to COVID-19 infection. We hope this represents an engaged subset of patients and more will be convinced over time to accept the COVID-19 vaccine as well as COVID-19 booster vaccines so we can have a more robust vaccinated population to better combat this pandemic. This study illustrates the importance of physicians, and especially rheumatologists, discussing COVID-19 and the COVID-19 vaccine with their patients.

## Supplementary Information

**Additional file 1** Supplementary table.

## Data Availability

Data used and/or analyzed during the current study are available from the corresponding author on reasonable request.

## Abbreviations

COVID 19: Coronavirus disease
LAC + USC: Los Angeles County + University of Southern California
mRNA: Messenger Ribonucleic Acid
RD: Rheumatic disease
SARS-CoV-2: Severe acute respiratory syndrome coronavirus 2
SLE: Systemic Lupus Erythematosus
HCQ: Hydroxychloroquine
MTX: Methotrexate
