# Supplementary material for: Attitudes and beliefs towards COVID-19 and COVID-19 vaccination among rheumatology patients in a Los Angeles County safety net clinic
Source: BMC Rheumatol. 2023 Jun 1;7:13. doi: 10.1186/s41927-023-00338-7 (PMC10231953; doi:10.1186/s41927-023-00338-7)
Supplement: Supplementary file 1 — Additional file 1 Supplementary table. [file 41927_2023_338_MOESM1_ESM.docx]

|  |  | Non-hesitant group | Hesitant group | p-value |
| --- | --- | --- | --- | --- |
|  | Age |  |  |  |
|  | 18-40 | 17 | 2 | 0.87 |
|  | >40 | 82 | 11 |  |
|  |  |  |  |  |
|  | Education* |  |  |  |
|  | Some school | 68 | 9 | 0.78 |
|  | More than school | 27 | 3 |  |
|  |  |  |  |  |
|  | Personal experience with COVID-19* |  |  |  |
|  | Yes | 44 | 5 | 0.66 |
|  | No | 36 | 4 |  |
|  |  |  |  |  |

*missing data excluded from analysis

**Additional File 1:** Post -hoc sensitivity analysis of association of COVID-19 vaccine with key demographic factors.
